# Clinicopathological and Prognostic Roles of STAT3 and Its Phosphorylation in Glioma

**DOI:** 10.1155/2020/8833885

**Published:** 2020-11-22

**Authors:** Bo Liang, Shuang-Yang Li, Hui-Zhi Gong, Ling-Xue Wang, Jia Lu, Yu-Xiu Zhao, Ning Gu

**Affiliations:** ^1^Nanjing University of Chinese Medicine, Nanjing, China; ^2^Hospital (T.C.M.) Affiliated to Southwest Medical University, Luzhou, China; ^3^Nanjing Hospital of Chinese Medicine Affiliated to Nanjing University of Chinese Medicine, Nanjing, China

## Abstract

Glioma is defined as a common brain tumor which causes severe disability or death. As many genes are reported to relate with glioma's occurrence and development, their prognostic and therapeutic value still remains uncertain. This study aimed at investigating the association between STAT3/p-STAT3 and glioma prognosis. Nine studies (12 trials) scored ≥5 on the Newcastle-Ottawa scale were meta-analysed from the Medline, Embase, and Web of Science databases. We found that STAT3/p-STAT3 overexpression in glioma patients was associated with worse overall survival (hazard  ratio  (HR) = 1.40, 95%confidence  interval  (CI) = 1.05 ~ 1.86, *P* = 0.020), progression-free survival (HR = 2.05, 95%CI = 1.63 ~ 2.58, *P* < 0.001), and better recurrence-free survival (HR = 0.37, 95%CI = 0.15 ~ 0.95, *P* < 0.039). Subgroup analysis implied that STAT3/p-STAT3 overexpression was associated with worse OS in standard treatment (HR = 1.80, 95%CI = 1.06 ~ 3.04, *P* = 0.030), and in China (HR = 2.18, 95%CI = 1.77 ~ 2.70, *P* < 0.001), and metaregression analysis indicated countries (*P* = 0.001) may be the source of heterogeneity in our study. In conclusion, we suggested STAT3/p-STAT3 was associated with poor prognosis in patients with glioma, which indicated that STAT3/p-STAT3 might be a valuable prognostic biomarker and a promising therapeutic target for glioma.

## 1. Introduction

Brain malignancy is a grievous type of brain tumor with high incidence and mortality, while the concentration on brain malignancy is still poor [1, 2]. Glioma, starting in the glial cells of the brain or the spine [3], is the most common primary intracranial tumor, which represents about 30% of all brain tumors and central nervous system tumors, and 80% of all malignant brain tumors [2, 4]. Although the etiology of glioma remain unclear, researches on monogenic Mendelian disorders [4], hereditary genetic disorders such as neurofibromatoses (type 1 and type 2), and tuberous sclerosis complex underscore that genetic factors are strongly associated with the development and progression of glioma [5]. Results from genome-wide association studies have identified common genetic variation in 7 genes (*TERT* [6], *EGFR* [7, 8], *CCDC26* [9], *CDKN2B* [6], *PHLDB1* [10], *TP53* [11, 12], and *RTEL1* [6]) and germ-line (inherited) polymorphisms of the DNA repair genes *ERCC1*, *ERCC2*, and *XRCC1* increase the risk of glioma [13]. Recent study found that mutations in *IDH1/2* may result in the development of glioma and be independent prognosis factors of glioma [14]. These indicate altered or deficient repair of DNA damage and different oncogenes contribute to the development of glioma [15, 16]. Diet, radiation, and infection with cytomegalovirus are regarded as potential pathogenic factors [17, 18]. In addition, certain occupations, such as farmers, are more susceptible to this disease [19, 20]. The potential influences of occupational exposures and cell phones have also been examined, with inconclusive results [2].

Although glioma is relatively rare compared with cerebral vascular diseases, they both cause severe mortality and morbidity [21]. Symptoms of glioma depend on the brain region where the tumor locates and show as headaches, vomiting, seizures, and cranial nerve disorders as a result of mass effect and increased intracranial pressure. Although glioma treatment options have developed, the prognosis remains poor. Multiple genes have been identified as glioma biomarkers that may predict patient's susceptibility and prognosis [21]. STAT3 is a latent cytosolic transcription factor and activates genes in human chromosome 12 (q13 to q14-1) by phosphorylating the tyrosine705 in the SH2 domain [22]. STAT3 plays an important role in multiple malignant cases, especially in glioma [23–25]. Phosphorylated STAT3 (p-STAT3) dimerizes spontaneously, migrates into cell nucleus, and activates the expression of downstream genes to regulate the tumor cell growth, proliferation, differentiation, and metastasis [26, 27]. In addition, p-STAT3 was reported to affect the occurrence, development, and even prognosis of glioma [28–31]. Emerging studies have shown STAT3 and its phosphorylation have similar functions in tumors [32–34], including in glioma [35–37], and given this consideration, we conducted a meta-analysis of STAT3/p-STAT3 to identify any direct correlations with glioma patient prognosis.

## 2. Materials and Methods

### 2.1. Study Search Strategy

We comprehensively searched for potential studies from the Medline, Embase, and Web of Science databases using relevant key words through December 1, 2018, without any language restrictions. The detailed literature search strategy in Medline was ((STAT3 Transcription Factor [Mesh]) OR (((((((APRF Transcription Factor) OR (Signal Transducer and Activator of Transcription 3)) OR IL6 Response Factor) OR LIF Response Factor) OR STAT3b Transcription Factor) OR STAT3a Transcription Factor) OR Transcription Factor, STAT3 [All fields]) OR ((((phosphorylated signal transducer and activator of transcription 3)) OR phosphorylated stat3 transcription factor) OR phospho-STAT3 [All fields])) AND ((Glioma [Mesh]) OR ((((((Glial Cell Tumors [All fields]) OR Glial Cell Tumor [All fields]) OR Mixed Glioma [All fields]) OR Tumor, Glial Cell [All fields]) OR Malignant Glioma [All fields]) OR Malignant Gliomas [All fields])).

### 2.2. Study Selection

Studies included in this analysis must meet some criteria. The participants must have been diagnosed with glioma via imaging, pathology, or the latest clinical diagnostic criteria. There was immunohistochemical analysis with the expression of STAT3/p-STAT3 in the glioma tissue. The association between STAT3/p-STAT3 and patients' prognosis, regardless overall survival (OS), progression-free survival (PFS), or recurrence-free survival (RFS), was investigated, and the adjusted or crude hazard ratio (HR) values could be calculated. Only studies with a sample size more than 60 were included. When the overlapping or even same data appears in different studies, the most complete or up-to-date study was included. The review and abstract were excluded [38].

### 2.3. Data Extraction

Two reviewers independently extracted the following data from remaining studies: the information about study characteristics (such as the first author, publication date, country, sample size, treatment, and analytic method), demographic characteristics (including age, sex, and diagnostic methods), the expression of STAT3/p-STAT3, and outcomes. Then, another two reviewers checked the received data. Inconsistent data were addressed by open discussion, and consensus was achieved. Finally, all extracted data were stored in the predesigned excel spreadsheet.

### 2.4. Study Quality Assessment

Two investigators conducted studies quality assessments of the included studies according to the Newcastle-Ottawa scale (NOS) developed for nonrandomized controlled trials [39]. The NOS included three domains: selection, comparability, and outcome, eight items with nine scores in total [40, 41].

### 2.5. Statistical Analysis

For all analyses, we used fixed and random effect models to reduce heterogeneity through Stata 14.0 (Stata Corp., College Station, TX, USA). Depending on the type of extracted data, we applied subgroup analysis by country, sample size, study subject (STAT3 or p-STAT3), treatment, and HR estimate. Moreover, sensitivity analysis was conducted to assess heterogeneity. We would assign adjectives of low, moderate, and high to *I*^2^ values of 25%, 50%, and 75% as described previously [38, 40, 42]. Lastly, the funnel plot was implemented to assess the publication bias and metaregression analysis was applied to trace the origin of heterogeneity [41].

## 3. Results

### 3.1. Study Search Results and Characteristics

**Figure 1:**
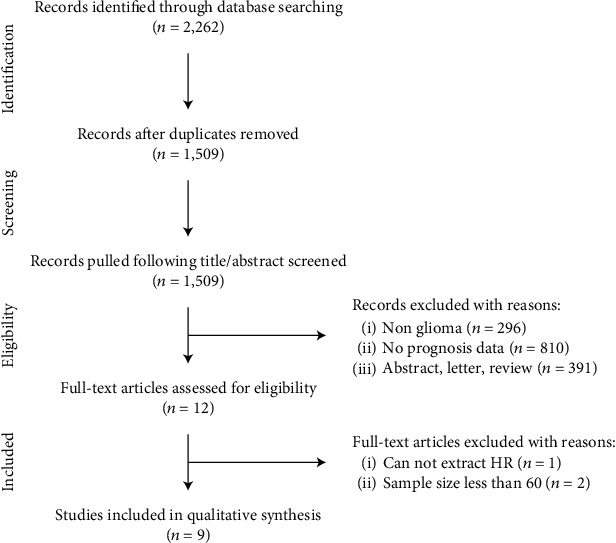
The flow chart.

The combined search yielded 1,509 potentially relevant studies after removing 753 duplicates, and 12 were retained while the title and abstract were screened. Three studies were close to meet the threshold but still excluded due to their lack of detailed data and sample size. Nine studies [43–51] (12 trials) were included in this study (Figure 1), among which 3 trials evaluated STAT3, and other three-fourths detected p-STAT3. As for countries, almost half of studies came from China [43–46, 51], and one each from Brazil [50], Greece [48], Bulgaria [47], and America [49] (Table 1). With the exception of one study (NOS  score = 5 [49], all other studies had NOS scores of 7 or above, which were considered a high quality (or low-bias risk) studies [40, 41] (Table 1).

### 3.2. Association of STAT3/p-STAT3 with OS

**Figure 2:**
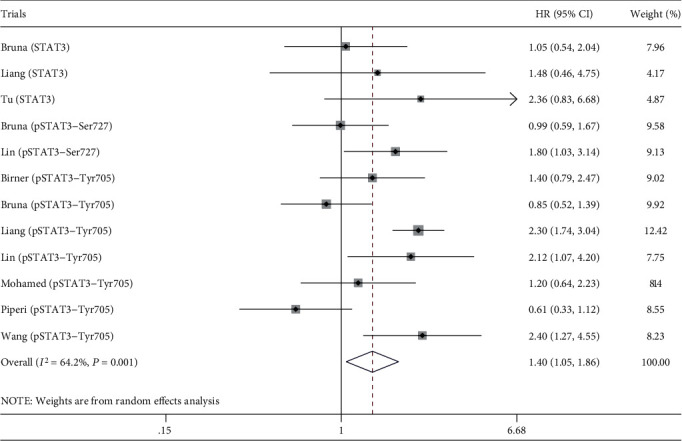
Association of STAT3/p-STAT3 with OS.

**Figure 3:**
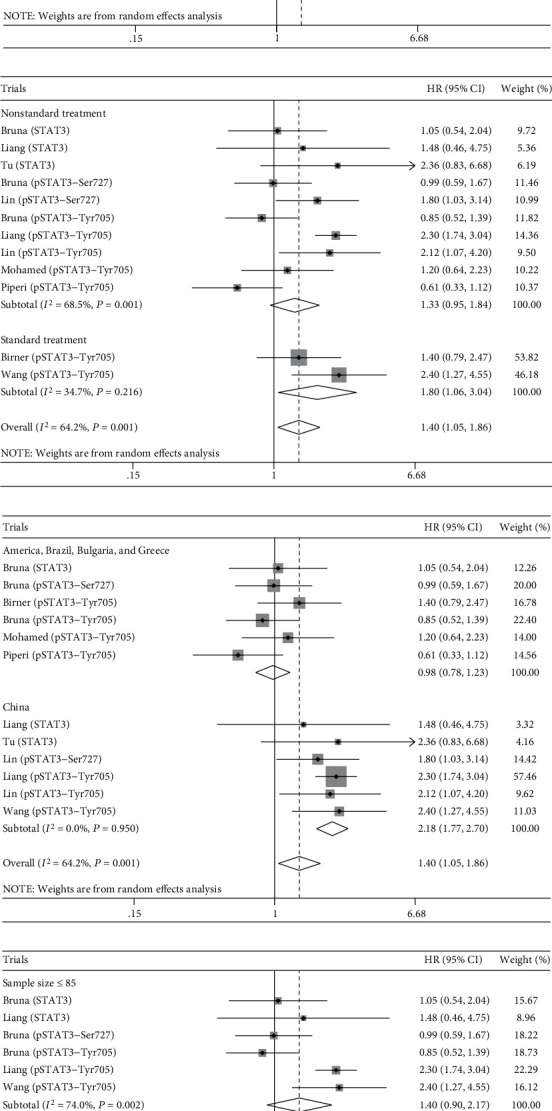
Subgroup analysis. (a) STAT3/p-STAT3. (b) Treatment. (c) Country. (d) Sample size.

The combined analysis of 12 trials showed that STAT3/p-STAT3 overexpression in glioma was associated with worse OS (HR = 1.40, 95% confidence interval (CI) = 1.05 ~ 1.86, *P* = 0.020) (Figure 2). Obviously, there was significant heterogeneity among trials (*I*^2^ = 64.2%, *P*_h_ = 0.001), so we conducted subgroup and metaregression analysis to investigate the possible source of the heterogeneity. As for STAT3 and its phosphorylation, there was no association with OS (Figure 3(a)). Furthermore, we conducted subgroup analysis of STAT3 phosphorylation site (Tyr705 and Ser727), and the results were consistent (Figure S1). Treatment subgroup analysis results implied that STAT3/p-STAT3 overexpression was associated with worse OS in standard treatment (HR = 1.80, 95%CI = 1.06 ~ 3.04, *P* = 0.030), and there was no significant association in nonstandard treatment (HR = 1.33, 95%CI = 0.95 ~ 1.84, *P* = 0.095) (Figure 3(b)). As for country subgroup analysis, STAT3/p-STAT3 overexpression was associated with worse OS in China (HR = 2.18, 95%CI = 1.77 ~ 2.70, *P* < 0.001), but no evidences in other countries (HR = 0.98, 95%CI = 0.78 ~ 1.23, *P* = 0.852) (Figure 3(c)). Subgroup analysis results of sample size showed that STAT3/p-STAT3 expression was not associated with OS (Figure 3(d)). Moreover, metaregression analysis indicated countries (*P* = 0.001) may be the source of heterogeneity in this study, while STAT3/p-STAT3 (*P* = 0.863), treatment (*P* = 0.423), and sample size (*P* = 0.996) were not.

### 3.3. Association of STAT3/p-STAT3 with PFS

**Figure 4:**
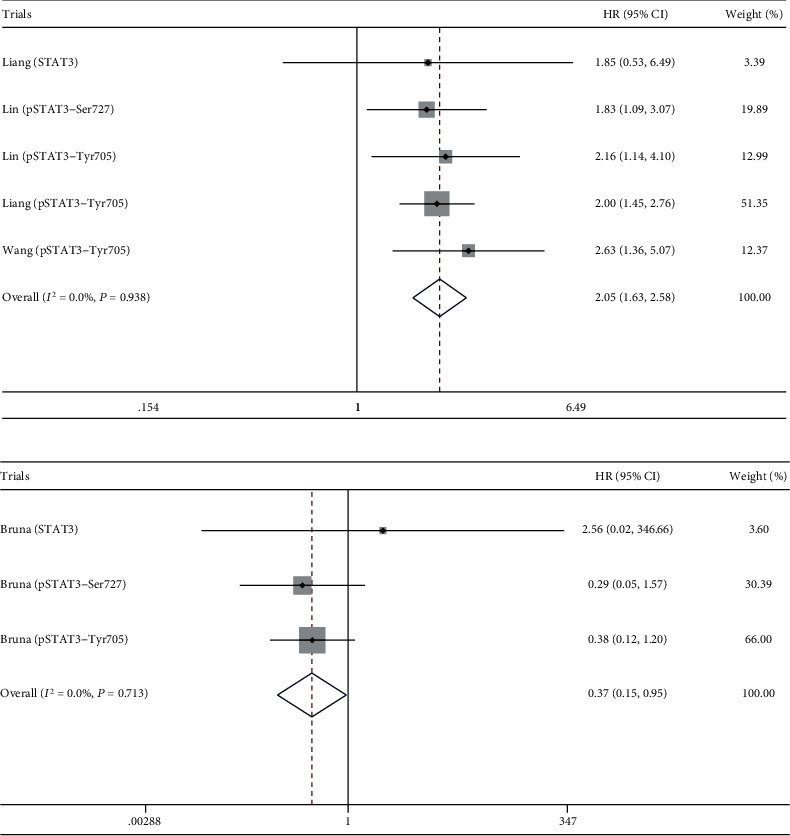
Association of STAT3/p-STAT3 with PFS and RFS. (a) PFS. (b) RFS.

The combined analysis of 5 trials suggested STAT3/p-STAT3 overexpression in glioma was associated with worse PFS (HR = 2.05, 95%CI = 1.63 ~ 2.58, *P* < 0.001) (Figure 4(a)). It is clear that there was no heterogeneity among trials (*I*^2^ = 0.0%, *P*_h_ = 0.938) (Figure 4(a)).

### 3.4. Association of STAT3/p-STAT3 with RFS

The combined analysis of 3 trials showed that STAT3/p-STAT3 overexpression in glioma was associated with better RFS (HR = 0.37, 95%CI = 0.15 ~ 0.95, *P* < 0.039) (Figure 4(b)). It is clear that there was no heterogeneity among trials (*I*^2^ = 0.0%, *P*_h_ = 0.713) (Figure 4(b)).

### 3.5. Sensitivity Analysis

**Figure 5:**
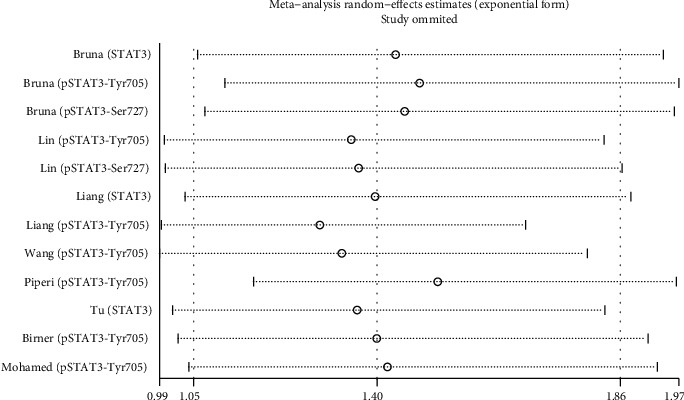
Sensitivity analysis.

We removed each trial and reanalysed the data, and the main findings were unchanged (Figure 5).

### 3.6. Publication Bias

**Figure 6:**
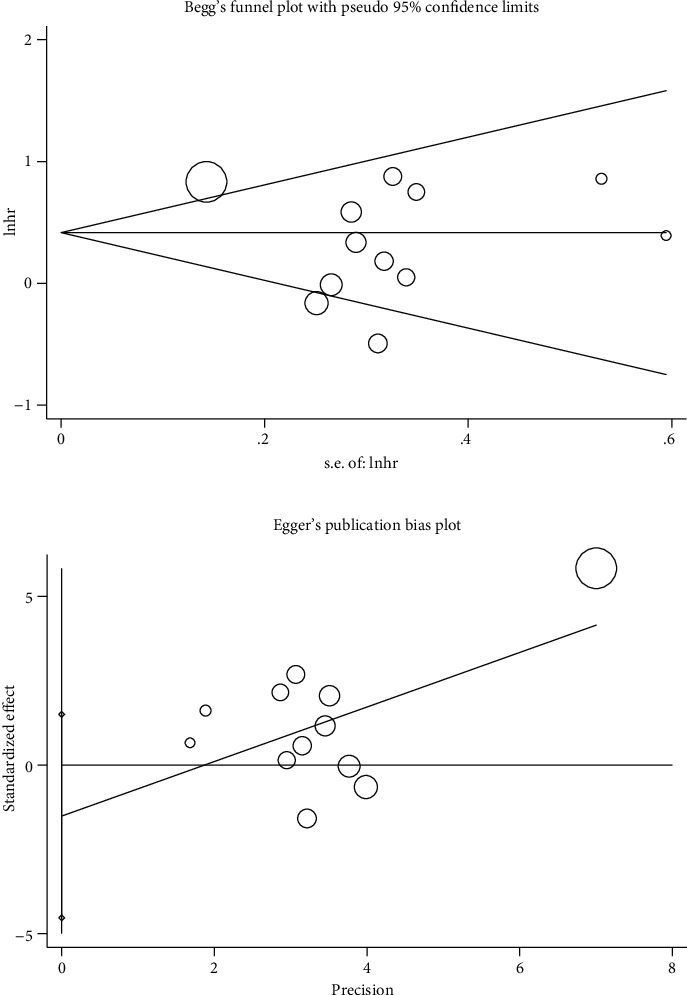
Publication bias.

Funnel plot analysis showed that there was no statistical evidence of publication bias in this study (*P*_Begg's  test_ = 0.631 and *P*_Egger's  test_ = 0.290, respectively) (Figure 6).

## 4. Discussion

Few known risk factors are associated with the brain and central nervous system cancer, including glioma [52], and the only consistent correlations resulted from epidemiological studies [53, 54]. In our knowledge, this study is the most comprehensive assessment of the trials regarding STAT3/p-STAT3 expression and glioma prognosis to date. We systematically evaluated survival data for 1,070 glioma patients included in 12 different trials and came to the conclusion that the expression of STAT3/p-STAT3 may be a marker of poor prognosis in glioma. Our study shows that STAT3/p-STAT3 expression is related to poor glioma prognosis. The results of subgroup analysis further emphasize the importance and effectiveness of standard treatment and it also highlights the potential of STAT3/p-STAT3 for the development of valuable prognostic biomarkers and therapeutic agents of glioma.

Our study also inevitably has some limitations. Summarized population-level data, rather than individual patient-level data, was only extracted from the included 9 studies. The expression and positive rate of STAT3/p-STAT3 were diverse in different studies while immunohistochemical analysis was used to detect STAT3/p-STAT3. In addition, the scope of our research results may be limited as most of included studies were from China, while our results indicated that STAT3/p-STAT3 overexpression was associated with worse OS in China. Unfortunately, this limitation cannot be improved, given the chosen inclusion criteria. Last of all, HR and 95% CI were not directly available in some included studies and we had to apply Engauge Digitizer to obtain them from the survival curve, and heterogeneity under such circumstances was so substantial that random effect models and subgroup analysis could not diminish it; thus, additional analysis is necessary to clarify these confusing problems. Glioma is classified by cell type, by grade, and by location, but we failed to conduct subgroup analysis of classification due to the limited data included in this study, as well as meta-analysis of RFS or PFS. Moreover, during the analysis, we combined STAT3 and p-STAT3 together to analyse. It is precisely for this reason that this study cannot be more in-depth and thorough.

In conclusion, our study suggested that STAT3/p-STAT3 is associated with poor prognosis in patients with glioma, which indicated that STAT3/p-STAT3 might be a valuable prognostic biomarker and a promising therapeutic target for glioma. Further studies with larger sample sizes and multicenter/countries are needed to shed more light on the more precise correlation between STAT3/p-STAT3 and glioma.

## Figures and Tables

**Table 1:** Main characteristics of all included studies.

Trials	Year	Country	Sample size	Treatment	HR for OS (95% CI)	HR for RFS (95% CI)	HR for PFS (95% CI)	NOS	References
Bruna (STAT3)	2016	Brazil	85	Nonstandard	1.050 (0.540-2.040)	2.560 (0.006-110.040)	NA	8	[50]
Bruna (pSTAT3-Tyr705)	2016	Brazil	85	Nonstandard	0.850 (0.520-1.390)	0.380 (0.120-1.190)	NA	8	[50]
Bruna (pSTAT3-Ser727)	2016	Brazil	85	Nonstandard	0.990 (0.590-1.670)	0.290 (0.050-1.470)	NA	8	[50]
Lin (pSTAT3-Tyr705)	2014	China	90	Nonstandard	2.120 (1.070-4.203)	NA	2.158 (1.136-4.098)	8	[44]
Lin (pSTAT3-Ser727)	2014	China	88	Nonstandard	1.797 (1.028-3.142)	NA	1.830 (1.090-3.074)	9	[45]
Liang (STAT3)	2013	China	68	Nonstandard	1.480 (0.460-4.730)	NA	1.850 (0.530-6.520)	9	[51]
Liang (pSTAT3-Tyr705)	2013	China	68	Nonstandard	2.301 (1.726-3.021)	NA	2.001 (1.509-2.877)	9	[51]
Wang (pSTAT3-Tyr705)	2011	China	68	Standard	2.402 (1.268-4.550)	NA	2.629 (1.362-5.073)	9	[46]
Piperi (pSTAT3-Tyr705)	2011	Greece	97	Nonstandard	0.611 (0.332-1.124)	NA	NA	9	[48]
Tu (STAT3)	2011	China	96	Nonstandard	2.360 (1.370-10.980)	NA	NA	7	[43]
Birner (pSTAT3-Tyr705)	2010	Bulgaria	111	Standard	1.400 (0.790-2.460)	NA	NA	7	[47]
Mohamed (pSTAT3-Tyr705)	2008	America	129	Nonstandard	1.200 (0.640-2.220)	NA	NA	5	[49]

Note: HR: hazard ratio; OS: overall survival; RFS: recurrence-free survival; PFS: progression-free survival; NOS: Newcastle-Ottawa scale; NA: not applicable.

## Data Availability

All data generated or analysed during this study are included in this published article and its supplementary information files.

## Supplementary Materials

Supplementary Materials Figure S1: subgroup analysis of STAT3 phosphorylation site.
